# Hyaluronan concentration and size distribution in human knee synovial fluid: variations with age and cartilage degeneration

**DOI:** 10.1186/s13075-016-0922-4

**Published:** 2016-01-21

**Authors:** Michele M. Temple-Wong, Shuwen Ren, Phu Quach, Bradley C. Hansen, Albert C. Chen, Akihiko Hasegawa, Darryl D. D’Lima, Jim Koziol, Koichi Masuda, Martin K. Lotz, Robert L. Sah

**Affiliations:** Department of Bioengineering, University of California-San Diego, 9500 Gilman Drive, Mail Code 0412, La Jolla, CA 92093-0412 USA; Department of Molecular and Experimental Medicine, MEM-161, The Scripps Research Institute, 10550 North Torrey Pines Road, La Jolla, CA 92037 USA; Shiley Center for Orthopaedic Research & Education at Scripps Clinic, 10550 North Torrey Pines Road, La Jolla, CA 92037 USA; Department of Orthopaedic Surgery, University of California-San Diego, 9500 Gilman Drive, Mail Code 0412, La Jolla, CA 92093-0412 USA; Center for Musculoskeletal Research, Institute of Engineering in Medicine, University of California-San Diego, 9500 Gilman Drive, Mail Code 0412, La Jolla, CA 92093-0412 USA

**Keywords:** Hyaluronan, Synovial fluid, Aging, Degeneration

## Abstract

**Background:**

One potential mechanism for early superficial cartilage wear in normal joints is alteration of the lubricant content and quality of synovial fluid. The purpose of this study was to determine if the concentration and quality of the lubricant, hyaluronan, in synovial fluid: (1) was similar in left and right knees; (2) exhibited similar age-associated trends, whether collected postmortem or antemortem; and (3) varied with age and grade of joint degeneration.

**Methods:**

Human synovial fluid of donors (23–91 years) without osteoarthritis was analyzed for the concentrations of protein, hyaluronan, and hyaluronan in the molecular weight ranges of 2.5–7 MDa, 1–2.5 MDa, 0.5–1 MDa, and 0.03–0.5 MDa. Similarity of data between left and right knees was assessed by reduced major axis regression, paired *t*-test, and Bland-Altman analysis. The effect of antemortem versus postmortem collection on biochemical properties was assessed for age-matched samples by unpaired *t*-test. The relationships between age, joint grade, and each biochemical component were assessed by regression analysis.

**Results:**

Joint grade and the concentrations of protein, hyaluronan, and hyaluronan in the molecular weight ranges of 2.5–7 MDa, 1–2.5 MDa, and 0.5–1 MDa in human synovial fluid showed good agreement between left and right knees and were similar between age-matched patient and cadaver knee joints. There was an age-associated decrease in overall joint grade (–15 %/decade) and concentrations of hyaluronan (–10.5 %/decade), and hyaluronan in the molecular weight ranges of 2.5–7 MDa (–9.4 %/decade), 1–2.5 MDa (–11.3 %/decade), 0.5–1 MDa (–12.5 %/decade), and 0.03–0.5 MDa (–13.0 %/decade). Hyaluronan concentration and quality was more strongly associated with age than with joint grade.

**Conclusions:**

The age-related increase in cartilage wear in non-osteoarthritic joints may be related to the altered hyaluronan content and quality of synovial fluid.

## Background

Synovial fluid (SF) functions as a biological lubricant and biochemical pool of nutrients and regulatory cytokines. It reduces friction at cartilage–cartilage interfaces in the boundary mode of lubrication [1]. The early cartilage wear that occurs in macroscopically normal joints with aging and is evident at the superficial zone [2] suggests that the wear-protective function of SF and wear-resistance of cartilage are altered with aging and may lead to the development of osteoarthritis (OA). One possible mechanism for these changes in the knee is age- and disease-related deterioration of SF lubricant quality. Such diminished SF lubrication has been observed following acute injuries in horses [3] and humans [4], and in experimental models of osteoarthritis in rabbits [5] and guinea pigs [6]. The elucidation of situations in which SF lubrication properties and lubricant biomolecules are diminished could lead to the development of treatments to correct such lubrication deficiency.

A primary lubricant molecule in SF is hyaluronan (HA), interacting with and adsorbing to the articular surface [7]. HA is present in SF as a polydisperse polymer of repeating disaccharides of N-acetyl-glucosamine and glucuronic acid, connected exclusively by β-linkages of up to 20 MDa [8]. In the boundary mode of lubrication, the cartilage-on-cartilage (but not cartilage-on-glass [9] or mica-on-mica [10]) friction-lowering properties of HA are dependent on its concentration [11] and molecular mass (M_r_) [3], with lubrication properties being better for higher-M_r_ forms of HA. The HA concentration in human SF (hSF) ranges widely, from 1 to 4 mg/ml [12, 13]. Few studies have described the age-related variations in HA concentration or size in hSF. hSF HA concentration has been reported as changing little with age or tending to decrease between 28 and 40 years of age and remaining at a low level beyond that age [13, 14], with little known about the possible effects of age- and OA-related degeneration. In OA compared to normal knees, the HA concentration is lower [12, 15], with a shift to lower M_r_ forms of HA [12, 16]. However, the age-associated changes in the content and M_r_ of HA in hSF from knee joints without OA are unclear.

The hypothesis tested in this study was that the HA concentration and M_r_ distribution in hSF fluid varies with adult age and cartilage degeneration. Thus, the aims of this study were to determine if hSF concentration of HA as well as protein, and M_r_ distribution of HA: (1) differed between left and right knees of individual donors; (2) differed between cadaveric donors and patients; and varied with (3) age and (4) grade of joint degeneration.

## Methods

### Study design

To address the four aims, hSF was analyzed from knee joints without OA. hSF was aspirated from one or both knee joints (n = 48 joints) of cadaveric human donors (n = 28 donors) within 72 hours of death. The donors were without OA based on exclusion criteria of a history of knee arthritis or trauma, body mass index (BMI) >30 kg/m^2^, and macroscopic evidence of osteophytes, full-thickness erosion, or severe degeneration. Donors were selected to reflect an approximately even distribution of age and gender, with age from 23–91 years (61 ± 19 years, mean ± standard deviation (SD)), 12 male and 16 female donors, and BMI of 12–30 kg/m^2^ (25 ± 8 kg/m^2^). hSF was obtained within 72 hours of death (48 ± 15 hours). In addition, data were obtained about hSF obtained previously in vivo from consenting patients of an Institutional Review Board approved study at the University of California, San Diego [4], from non-injured and non-OA knees of a subset of patients that ranged in age from 25–59 years (45 ± 15 years) and included six male and two female donors.

Each of these hSF samples was analyzed for the concentrations of protein (c_Pro_), HA (c_HA_) and HA in M_r_ ranges of 2.5–7 MDa (c_HA(2.5–7MDa)_), 1–2.5 MDa (c_HA(1–2.5MDa)_), 0.5–1 MDa (c_HA(0.5–1MDa)_) and 0.03–0.5 MDa (c_HA(0.03–0.5MDa)_).

The similarity between left and right knees of overall joint grade and hSF properties was analyzed by reduced major axis regression (with one delete jack-knife estimates of the slope, intercept, regression coefficient, and associated standard errors) [17, 18], paired t-tests, and Bland-Altman analysis [19].

To determine how hSF compared between cadaver donor and patient knees, age-matched patient and cadaver hSF samples were compared. The effect of hSF source (patient vs. cadaver) on c_Pro_, c_HA_, c_HA(2.5–7MDa)_, c_HA(1–2.5MDa)_, c_HA(0.5–1MDa)_, and c_HA(0.03–0.5MDa)_ of age-matched hSF samples was assessed by analysis of variance with age as a covariate.

The effect of age and stage of joint degeneration on the concentrations of protein and HA and HA M_r_ distribution were assessed. Because of the similarity of several HA properties between left and right knees, data from left and right knees were not treated as independent measures, and further analyses were performed on the average of data from left and right knees of the same donor. Also, due to the similarity between patient and cadaver hSF samples, data from patient and cadaver hSF were analyzed together to assess the effect of age and joint degeneration on biochemical measures. Univariate linear regression was performed to assess the effect of age on joint grade as well as the effect of age on each biochemical measure and the effect of joint grade on each biochemical measure. To further assess the effect of age on the fraction of HA in each M_r_ bin, a beta regression model was applied [20]. To assess whether each HA biochemical property was dependent on both joint grade and age, multivariate regression with the backward elimination procedure was performed.

### Macroscopic joint grading

Joints were screened by macroscopic observation. An overall joint grade was determined for each cadaver knee joint as a sum of modified Outerbridge macroscopic grades (1–4) of each of nine regions of each condyle, three regions on the trochlea, nine regions of each tibial plateau, and nine regions on the patella, as described by the International Cartilage Repair Society (ICRS) [21, 22]. ICRS scores can thus range from 48 for normal knees to 192 for degenerate knees. Joints with severe degeneration (grade of 96 or greater) were excluded, as were joints where subchondral bone was exposed by full-thickness cartilage erosion.

### Preparation of synovial fluid

For both cadaver and patient knees, the hSF was aspirated using a standard 18-gauge hollow bore needle attached to a 10 cc or 60 cc syringe. Synovial fluid was clarified of cells and debris by centrifugation at 3000 g for 30 minutes at 4 °C and stored at –70 °C until use.

### Biochemical analysis of synovial fluid

Portions of hSF were assayed for the concentrations of total protein and HA, as well as the M_r_ distribution of HA.

#### Protein concentration

Total protein was quantified with the BCA assay (Thermo Fisher Scientific Inc, Rockford, IL, USA). A portion of SF was diluted 1:30 in water and assayed following the manufacturer’s protocol. In pilot studies, the background absorbance of SF samples at that dilution (with water instead of working reagent) corresponded to an apparent protein concentration of 0.29 ± 0.01 mg/ml (mean ± standard error of the mean, n = 30 samples). Since this was negligible (<2 %) relative to the typical protein concentrations of SF (17 mg/ml), background was not routinely assessed.

#### HA concentration

The concentration of HA was determined by an enzyme-linked immunosorbent assay-like assay using recombinant human aggrecan for detection (R&D Systems, Minneapolis, MN, USA) following digestion of the protein component with proteinase K (0.5 mg/ml) overnight at 37 °C and inhibition of proteinase K by heating to 100 °C for 10 minutes. According to the manufacturer’s protocol, the assay detects HA M_r_ forms as small as 15–40 kDa.

#### HA M_r_

M_r_ distribution of HA was determined using an agarose gel electrophoresis technique [23]. Briefly, hSF was treated with proteinase K and then heating. Sample portions with HA mass of 200–500 ng were applied to 1 % agarose gels (Lonza, Rockland, ME, USA), separated by horizontal electrophoresis at 100 V for 110 minutes in TAE buffer (0.4 M Tris-acetate, 0.01 M EDTA, pH 8.3), and visualized by incubation with 0.1 % Stainsall reagent (Sigma, St. Louis, MO, USA). Gels were imaged, and images were analyzed by comparison to M_r_ standards to determine HA M_r_ distribution [24]. The proportions of HA within the M_r_ ranges of 0.03–0.5 MDa, 0.5–1.0 MDa, 1.0–2.5 MDa, and 2.5–7.0 MDa were calculated. The selection of M_r_ bins was based on the logarithmic relationship between HA M_r_ and electrophoretic mobility and to encompass the large range of HA M_r_ [23]. The concentration of HA within each M_r_ range was calculated as the proportion of HA in that range multiplied by the overall HA concentration. Since the molecular weight distribution of HA in SF is typically graded [23], the exact choice of HA M_r_ bins would not be expected to affect the main results, and we chose the bins used in our previous studies [3, 25].

## Results

### hSF variation between left and right knees

**Fig. 1:**
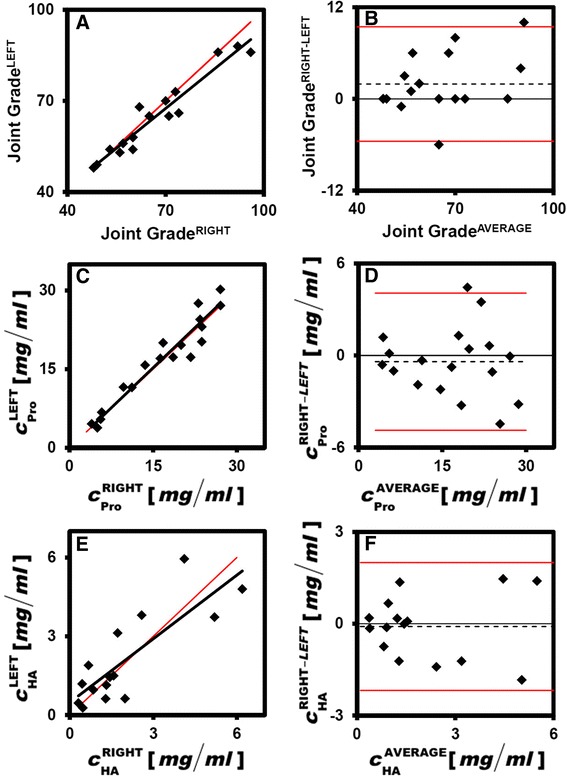
Graphical representation of left and right joint similarity. Data for left (*y axis*) and right (*x axis*) knees are plotted for joint grade (**a**), concentration of protein (*c*_*Pro*_) (**c**), and concentration of hyaluronan (*c*_*HA*_) (**e**); the *red line* represents the line of perfect concordance while the *black line* is the reduced major axis of the data. The difference between left and right knees is plotted against the average of left and right knees for joint grade (**b**), c_Pro_ (**d**), and c_HA_ (**f**); *dashed lines* represent the mean difference while *red lines* represent ± two standard deviations of the individual differences. n = 15–18 donors

Left and right knees from the same donor showed a number of similarities. Overall joint grades were low (mean = 65) and similar between right and left knees, with a slope of the reduced major axis approaching one (Table 1, Fig. 1a), a high regression coefficient (R^2^ = 0.94; Table 1), lack of a significant difference in paired t-tests (*p* = 0.5; Table 1) and most differences between left and right data points falling within the 95 % limits of agreement in Bland-Altman plots (Fig. 1b). Additionally, hSF HA properties, c_Pro_, c_HA_, c_HA(2.5–7MDa)_, c_HA(1–2.5MDa)_ and c_HA(0.5–1MDa)_, were similar between right and left knees (Table 1), with slopes of the reduced major axis approaching one, high regression coefficients (R^2^ = 0.65–0.95), most paired t-tests being insignificant (*p* = 0.3–0.9), and most left and right data points falling within the 95 % limits of agreement. HA was mostly in higher M_r_ fractions, with c_HA(0.03–0.5MDa)_ being low (mean = 0.02 mg/ml). Because of the high degree of similarity between data from left and right joints, subsequent analyses were performed on data averaged from left and right knees when both were available.

**Table 1:** Agreement between properties of left and right knees of donors for overall joint grade, and hSF concentrations of protein, hyaluronan, and hyaluronan in molecular weight ranges of 2.5–7 MDa, 1–2.5 MDa, 0.5–1 MDa, and 0.03–0.25 MDa

Measure	Mean ± SD	Slope	Intercept	R^2^	*p*	n
Overall joint grade	65 ± 14	0.89 ± 0.07	5 ± 4	0.94	0.5	18
c_Pro_	17 ± 8 mg/ml	1.02 ± 0.06	0.1 ± 1.0 mg/ml	0.95	0.45	18
c_HA_	2.2 ± 1.6 mg/ml	1.0 ± 0.2	0.2 ± 0.4 mg/ml	0.67	0.74	15
c_HA(2.5–7MDa)_	1.6 ± 1.2 mg/ml	1.0 ± 0.1	0.0 ± 0.3 mg/ml	0.65	0.51	15
c_HA(1–2.5MDa)_	0.4 ± 0.4 mg/ml	0.9 ± 0.2	0.03 ± 0.06 mg/ml	0.69	0.88	15
c_HA(0.5–1MDa)_	0.05 ± 0.07 mg/ml	0.6 ± 0.3	0.01 ± 0.01 mg/ml	0.65	0.27	15
c_HA(0.03–0.5MDa)_	0.02 ± 0.03 mg/ml	1.1 ± 0.2	0.013 ± 0.007 mg/ml	0.88	0.04	15

The mean and standard deviation (SD) of donor averaged left and right values, slope (mean ± standard error of the mean (SEM)), intercept (mean ± SEM), and correlation coefficient (R^2^) from reduced major axis regression, *p*-value from paired t-tests and number of samples (n) are shown. *C*_*Pro*_ Concentration of protein, *c*_*HA*_ Concentration of hyaluronan

### Similarity of age-matched patient and cadaver hSF properties

Properties of hSF were similar between samples from age-matched patient and cadaver knee joints. The age-matched samples had mean and SD of age that were similar (*p* = 0.84) for patient (44.9 ± 15.1 years (25–59 years) and cadaver (45.9 ± 13.3 years (23–62 years)) samples. hSF from patient and cadaver knee joints were similar in c_Pro_ (19 ± 9 mg/ml; *p* = 0.23), c_HA_ (2.3 ± 1.6 mg/ml; *p* = 0.20), c_HA(2.5–7MDa)_ (1.8 ± 1.3 mg/ml; *p* = 0.53), and c_HA(1–2.5MDa)_ (0.44 ± 0.37 mg/ml; *p* = 0.16), with a strong association with age (each *p* < 0.05). At smaller HA M_r_, hSF concentrations were relatively low, with patient c_HA(0.5–1MDa)_ (0.16 ± 0.08 mg/ml) and c_HA(0.03–0.5MDa)_ (0.10 ± 0.08 mg/ml) being much lower than concentrations of c_HA_, c_HA(2.5–7MDa)_, and c_HA(1–2.5MDa)_. Relative to patient hSF, cadaver hSF exhibited even lower c_HA(0.5–1MDa)_ (–65 %, *p* < 0.005) and c_HA(0.03–0.5MDa)_ (–52 %, *p* < 0.05).

### Relationships of hSF properties with age

**Fig. 2:**
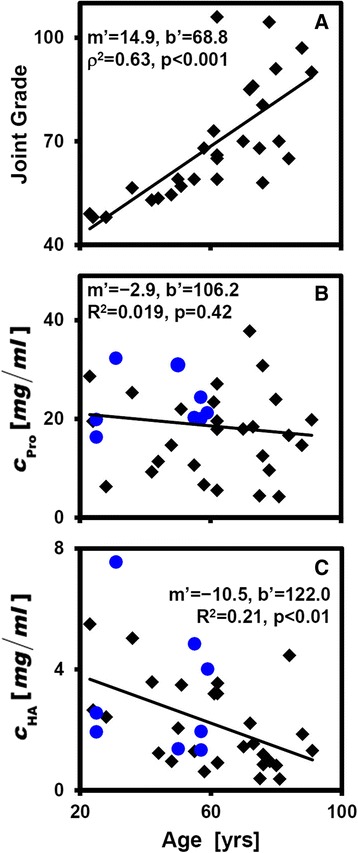
Relationships with age of overall joint grade (**a**), concentration of protein (*c*_*Pro*_) (**b**), and concentration of hyaluronan (*c*_*HA*_) (**c**). Data from cadaver (*black diamond*) and patient joints (*blue circles*) are shown. Slope (in percent per decade of age) and intercept (percent) of the best-fit line in the form of $100y/y(21)=m\hbox{'}\left(x/10\right)+b\hbox{'}$, the coefficient of determination (R^2^), and significance of the regression are shown. Because of the agreement between left and right knees, statistical analyses were performed using the average of data from left and right knees from each donor, if available. n = 28–36 donors

**Fig. 3:**
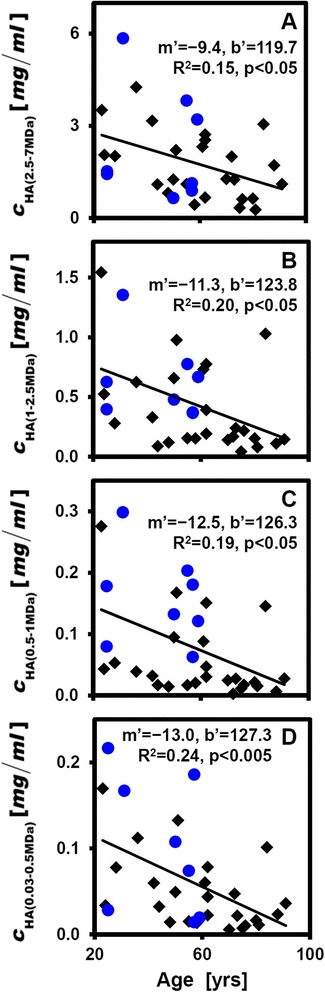
Relationships with age of c_HA(2.5–7MDa)_ (**a**), c_HA(1–2.5MDa)_ (**b**), c_HA(0.5–1MDa)_ (**c**), and c_HA(0.03–0.5MDa)_ (**d**). Data are shown as described in Fig. 1. n = 33. *c*_*HA*_ Concentration of hyaluronan

**Fig. 4:**
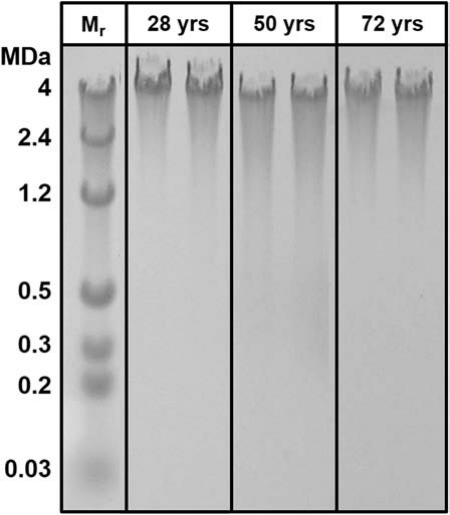
Electrophoretic pattern of HA in duplicate hSF samples from a 28-year-old donor, 50 year-old-donor, and 72-year-old donor. *M*_*r*_ Molecular weight mass

Overall joint grade and hSF HA content exhibited strong relationships with age. Univariate linear regression revealed strong associations with age of joint grade and certain hSF biochemical properties. Joint grade was strongly associated with age (R^2^ = 0.52; *p* < 0.001), increasing by ~15 % per decade of age (Fig. 2a). c_Pro_ was not related to age (*p* = 0.42; Fig. 2b) or joint grade (*p* = 0.62; Table 2). c_HA_ had a strong relationship with age (*p* < 0.01; Fig. 2c), decreasing ~10.5 % per decade of age, as did the concentration of HA in all M_r_ bins (c_HA(2.5–7MDa)_, c_HA(1–2.5MDa)_, c_HA(0.5–1MDa)_ and c_HA(0.03–0.5MDa)_), decreasing with age at rates of −9.4 to −13 % per decade of age (Fig. 3). Consistent with this, the distribution of HA was similar with age (e.g., Fig. 4) and the beta regression analysis did not detect a relationship to age of the fraction of HA in each M_r_ bin (*p* = 0.18–0.24). While there was a trend toward association of hSF HA and joint grade, the relationship did not achieve statistical significance (*p* = 0.17; Table 2), nor did M_r_ forms of HA (*p* = 0.21–0.48; Table 2). Multivariate regression confirmed the results of univariate linear regression.

**Table 2:** Relationships with overall joint grade of the quantities as described in Table 1

Measure	Slope (mg/ml)	R^2^	*p*	n
c_Pro_	0.051	0.01	0.62	28
c_HA_	–0.022	0.07	0.17	28
c_HA(2.5–7MDa)_	–0.013	0.04	0.35	25
c_HA(1–2.5MDa)_	–0.005	0.05	0.29	25
c_HA(0.5–1MDa)_	–0.0006	0.02	0.48	25
c_HA(0.03–0.5MDa)_	–0.0007	0.07	0.21	25

Because of the agreement between left and right knees, statistical analyses were performed using the average of data from left and right knees from each donor, if available. *C*_*Pro*_ Concentration of protein, *c*_*HA*_ Concentration of hyaluronan

## Discussion

The results of the present study delineated characteristics of HA in hSF from knees that exhibited early-stage age-associated deterioration but not OA. HA concentration was strikingly similar between left and right knees of the same cadaveric donor (R^2^ = 0.67; Fig. 1c, Table 1) and decreased substantially with age (R^2^ = 0.21; Fig. 2c). HA concentration in each of the M_r_ ranges studied here (c_HA(2.5–7MDa)_, c_HA(1–2.5MDa)_, c_HA(0.5–1MDa)_, and c_HA(0.03–0.5MDa)_; Fig. 3), decreased an average of ~11.3 % per decade of age (R^2^ = 0.15–0.24). Joint grade of the cadaveric joints (Fig. 2a) also varied with age, increasing ~10.5 % per decade of age. There was also an inverse association between hSF HA concentration and macroscopic joint grade, although this did not achieve statistical significance (*p* = 0.17) and the R^2^ was low (0.07).

The assessment of the natural variation in HA concentration and M_r_ distribution in knee hSF with age, in the absence of OA, can be affected by a number of factors. The similarity between donor left and right knees in joint grade, as well as HA biochemical properties, c_HA_, c_HA(2.5–7MDa)_, c_HA(1–2.5MDa)_, and c_HA(0.5–1MDa)_, and c_Pro_, relative to the variation between donors (Table 1) indicated that measures from paired knees should not be considered independent measures. In addition, the collection of cadaveric knee hSF 24–72 hours after death may reflect differences from the in vivo state, although concentration of hSF HA overall and in high M_r_ (>1 MDa) fractions was indistinguishable between age-matched samples from patients and cadaveric donors. Between the time of death and sample collection, concentrations of lower M_r_ HA may have diminished due to local diffusion or exudation of those solutes. However, the consistencies in hSF properties between age-matched patient and cadaver samples, as well as the between left and right knees of cadaver samples, suggest that the sampling method employed here provides an appropriate indicator of knee hSF status.

The age-related variation in hSF HA and protein concentrations determined here are consistent within and extend those reported previously. The hSF HA concentrations (2.2 ± 1.7 mg/ml) are within the published range for normal knees (0.8–3.8 mg/ml) [13, 14, 26]. The hSF protein concentrations (17 ± 8 mg/ml) are within range of that reported previously for normal knees (12–30 mg/ml) [26, 27] and were not age-dependent, consistent with the joints not being arthritic or injured. The overall decrease in HA concentration with age agrees with most studies [14, 28, 29] but not a report that did not observe a difference between 28–35 years and 52–78 years of age although viscosity properties did shift [13]. These differences may be due to the possible confounding effects of cartilage degeneration and OA disease, donor age distribution of samples selected for the studies, or differences in the sensitivity or type of biochemical methods employed (determination of hexosamine and hexuronic acid [13, 14] vs. immunochemical assay).

The similar age-associated decrease in concentration of HA in each of the analyzed M_r_ ranges is a new finding that may be particularly relevant to joint mechanics and mechanobiology. The decrease in high-M_r_ (1–2.5 MDa and 2.5–7 MDa) HA may be especially important, since these large molecules contribute primarily to boundary lubrication at articular cartilage–cartilage interfaces [3, 30] where HA may interact with PRG4 [10]. The age-related changes may be due to a variety of factors including HA anabolism, catabolism, and transport. The lack of variation of M_r_ distribution of hSF HA with age, reflecting integrative anabolic, catabolic, and transport effects, is distinct from effects of injury [31], where high M_r_ HA is selectively lost from SF. Histological study of normal synovial membrane showed no age-dependent changes in frequency of subsynovial blood vessel cross-sections, synovial intimal cells, mast cells, and subsynovial connective tissue cells [32]. On the other hand, HA degradation by hyaluronidases and reactive oxygen species generally result in HA of low M_r_ (0.8–20 kDa) [33, 34] that are rapidly lost from the joint [35] and can be angiogenic, inflammatory, and immunostimulatory [36]. The age-related decrease in high M_r_ HA with age may provide a target for modulation to restore hSF to young, normal lubricant molecule concentrations.

## Conclusions

The concentration decrease of HA in hSF with age, in the absence of OA, and the association of lower HA in SF with increased friction between cartilage surfaces, suggest that this relationship may be an important factor in the age-related deterioration of knee articular cartilage.

## Declarations

### Ethics approval and consent to participate

Human subjects included in this study were consenting patients in an Institutional Review Board (IRB) approved study at UCSD (IRB# 071788).

### Consent for publication

Not applicable.

### Availability of data and materials

Data are available upon request from the corresponding author.

## Abbreviations

BMI: Body mass index
c_HA_: Concentration of hyaluronan
c_Pro_: Concentration of protein
HA: Hyaluronan
hSF: Human synovial fluid
ICRS: International Cartilage Repair Society
M_r_: Molecular mass
OA: Osteoarthritis
SD: Standard deviation
SF: Synovial fluid
SEM: Standard error of the mean
